# Differential Enzymatic Activity of Rat ADAR2 Splicing Variants Is Due to Altered Capability to Interact with RNA in the Deaminase Domain

**DOI:** 10.3390/genes9020079

**Published:** 2018-02-08

**Authors:** Alice Filippini, Daniela Bonini, Edoardo Giacopuzzi, Luca La Via, Fabrizio Gangemi, Marina Colombi, Alessandro Barbon

**Affiliations:** 1 Division of Biology and Genetics-Department of Molecular and Translational Medicine, University of Brescia, Viale Europa 11, 25123 Brescia, Italy; a.filippini012@unibs.it (A.F.); daniela.bonini@unibs.it (D.B.); edoardo.giacopuzzi@unibs.it (E.G.); luca.lavia@unibs.it (L.L.V.); marina.colombi@unibs.it (M.C.); 2 Division of Physics, Department of Molecular and Translational Medicine, University of Brescia, Viale Europa 11, 25123 Brescia, Italy; fabrizio.gangemi@unibs.it

**Keywords:** ADAR2, RNA editing, alternative splicing, neuron

## Abstract

In mammals, adenosine (A) to inosine (I) RNA editing is performed by adenosine deaminases acting on RNA (ADAR), ADAR1 and ADAR2 enzymes, encoded by mRNAs that might undergo splicing process. In rat, two splicing events produce several isoforms of ADAR2, called ADAR2a, ADAR2b, ADAR2e, and ADAR2f, but only ADAR2a and ADAR2b are translated into an active protein. In particular, they differ for ten amino acids located in the catalytic domain of ADAR2b. Here, we focused on these two isoforms, analyzing the splicing pattern and their different function during rat neuronal maturation. We found an increase of editing levels in cortical neurons overexpressing ADAR2a compared to those overexpressing ADAR2b. These results indicate ADAR2a isoform as the most active one, as reported for the homologous human short variant. Furthermore, we showed that the differential editing activity is not due to a different dimerization of the two isoforms; it seems to be linked to the ten amino acids loop of ADAR2b that might interfere with RNA binding, occupying the space volume in which the RNA should be present in case of binding. These data might shed light on the complexity of ADAR2 regulations.

## 1. Introduction

The molecular complexity of the cells is usually achieved through a variety of mechanisms such as transcriptional regulation, mRNA processing and post-translational modifications. Alternative splicing is one of the mechanisms that contribute mostly to amplify the information from a single gene. Furthermore, other mechanisms generate diverse transcriptomes from a static genome; among these, RNA editing plays a pivotal role [1]. In mammals, two types of RNA editing have been reported [2] and they both involve the deamination of a specific nucleotide in a double strand RNA (dsRNA). The most frequent form of RNA editing in mammals is the adenosine (A)-to-inosine (I) editing [3]. Recent studies estimate millions of A-to-I editing events, mostly located in the human Alu inverted repeated elements, since they usually might generate dsRNA structure and are present in the majority of human genes [4]. However, in a few but extremely important cases, the editing events might be located in a coding sequence where the I is read as a guanosine (G) by the ribosomes, changing the meaning of RNA codons and thus altering the amino acid sequence of the translated protein.

The enzymes catalyzing the editing reaction are the Adenosine Deaminases acting on RNA (ADAR), which are highly conserved across metazoan. In mammals, there are three members of the ADAR family, ADAR1, ADAR2 and ADAR3; they work as homodimers [5] and are characterized by different roles [6,7,8,9] and localization [10,11,12,13,14,15]. All these enzymes have common structural elements: the Z-DNA binding domains, the double strand binding domains (dsRBD) and the catalytic domains (for review, see [16]). Only ADAR1 and ADAR2 have a proven enzymatic activity, while ADAR3 seems to act as a inhibitors of the editing process [17,18].

**Figure 1:**
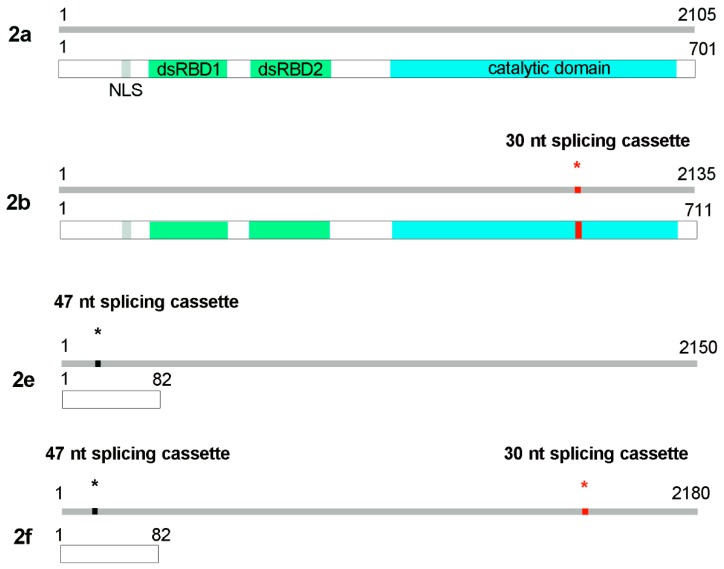
Rat adenosine deaminases acting on RNA (ADAR)2 mRNA and protein isoforms generated after alternative splicing. The scheme refers to NCBI RefSeq: ADAR2a NM_001111055.1, NP_001104525.1 (transcript and protein, respectively); ADAR2b NM_012894.2, NP_037026.2 (transcript and protein, respectively); and ADAR2e NM_001111057; ADAR2f NM_001111056.1. NLS: Nuclear Localization Signal; dsRBD: double strand RNA binding domain.

ADAR1 and ADAR2 transcripts undergo alternative splicing events, which give rise to several different protein isoforms. Concerning ADAR2, there are many splicing variants globally derived from two main splicing events [19], i.e., ADAR2a, ADAR2b, ADAR2e and ADAR2f (Figure 1).

In particular, the first splicing event, involving the 5′ end of the coding sequence, is due to a self-editing process that allows ADAR2 to act on its own pre-mRNA; in this way an alternative 3′ acceptor site is created converting an intronic AA to an AI, which mimics the AG dinucleotide, usually found in correspondence of a splicing site. This new acceptor site introduces in the transcript 47 nucleotides [19]; the new reading frame generates a stop codon in the mRNA and a truncated, inactive form of the protein is produced (ADAR2e and -2f) with 82 amino acids in rats and mice and 31 amino acids in humans. This self-editing mechanism is believed to have a role in controlling ADAR2 activity [20]. The second alternative splicing event involves the 3′ end of the coding sequence that is in the catalytic domain of the protein. Splicing at this site results in the inclusion of 30 nucleotides in rat and mouse (120 nucleotides in humans). The absence or the presence of this cassette generates the so-called ADAR2a or ADAR2b isoforms, respectively [21,22]. The last alternative splicing event is located at the end of the coding sequence and occurs only in humans, creating isoforms 2c and 2d [19,22]. This event implies the insertion of a shorter C terminus and the formation of an isoform without editing activity.

Several additional splicing events for ADAR2 were identified [23,24]. In human brain, a variant that skips exon 2 was found, resulting in a frameshift and in a premature stop codon, but the corresponding protein has not been detected. A second splicing variant creates two alternative 3′ UTR [24]. Moreover, a so-called exon 0 has been shown to extend the open reading frame by 49 amino acids at the N-terminus, but this ADAR2 isoform is not highly expressed [25].

In this work, we aimed to better characterize the two active rat splicing isoforms ADAR2a and ADAR2b; to this purpose, we explored the differential expression and RNA editing activity of these two variants, in order to understand how their differential modulation might affect RNA editing levels on specific targets.

## 2. Materials and Methods

### 2.1. Primary Cortical Cultures

Rat primary cortical cultures were prepared from Day 18 embryos, as previously described [26]. Briefly, cerebral cortices from embryos were mechanically dissociated in cold HBSS containing 10 mM HEPES (Invitrogen-ThermoFisher, Waltham, MA, USA); the cell suspension was re-suspended in serum-free Neurobasal medium (Invitrogen-ThermoFisher) supplemented with B-27 (Invitrogen-ThermoFisher), 30 U/mL penicillin (Sigma-Aldrich, St. Louis, MO, USA), 30 µg/mL streptomycin (Sigma-Aldrich), and 0.5 mM Glutamax (Invitrogen-ThermoFisher). The neurons were seeded at a density of 30,000 cells/cm^2^ on a poly-D-lysine coating (Sigma-Aldrich) in multi-well plates. Three days after plating, 50% of the medium was replaced with fresh medium; subsequently, half of the medium was replaced once a week for a maximum of four weeks.

### 2.2. RNA Extraction and Retrotranscription

Total RNA from cultured neurons was extracted using the ZymoResearchTM kit (Irvine, CA, USA), according to the manufacturer’s instructions. Briefly, 2 µg of total RNA were mixed with 2.2 µL of 0.2 ng/µL random hexamer (ThermoFisher, Waltham, MA, USA), 10 µL of 5× buffer (ThermoFisher), 10 µL of 2 mM dNTPs, 1 µL of 1 mM DTT (ThermoFisher), 0.4 µL of 33 U/µL RNasin (Promega, Madison, WI, USA), and 2 µL MMLV-RT (200 U/µL) in a final volume of 50 µL. The reaction mixture was incubated at 37 °C for 2 h, and then the enzyme was inactivated at 75 °C for 10 min.

### 2.3. RNA Editing Quantification

The editing levels for the target genes were quantified by sequence analysis as previously described [27,28]. Briefly, in the electropherogram obtained after real-time PCR (RT-PCR) and sequencing analysis of a pool of transcripts that might be edited or not, the nucleotide that undergoes the editing reaction appears as two overlapping peaks, A from unedited transcripts, and G from the edited ones. The editing level was calculated as a function of the ratio between the G peak area and A plus G peaks areas. The areas representing the amount of each nucleotide were quantified using Discovery Studio (DS) Gene 1.5 (Accelrys Inc., San Diego, CA, USA).

### 2.4. ADAR2 Splicing Isoforms Quantification

To evaluate the expression of the ADAR2 splicing isoforms, the first step was their selective amplification from cDNA samples; then, the Agilent 2100 bioanalyzer (Agilent, Santa Clara, CA, USA) allowed performing a capillary electrophoresis of the PCR products on specific chips through the Agilent DNA 1000™ kit, according to the manufacturer instructions.

The quantification of the splicing isoforms was performed through the ratio between the molarity of the −30/−47 band and the sum of the molarity of both the bands, i.e., (molarity ADAR2b/(molarity ADAR2b + molarityADAR2a) × 100).

### 2.5. Lentivirus Preparation

#### 2.5.1. Constructs

The replacement of the green fluorescent protein (GFP) sequence within the pRRLSIN.cPPT.PGK-GFP.WPRE with the HA-rADAR2a/Myc-rADAR2a sequence or HA-rADAR2b/Myc-rADAR2b was performed. The PCR reaction was set up using the Phusion High-Fidelity DNA Polymerase (F530S, 2 U/µL, ThermoFisher) in 50 µL of volume, according to the manufacturer instructions. The enzymatic digestion of 1 µg of both HA-rADAR2a/Myc-rADAR2a or HA-rADAR2b/Myc-rADAR2b and pRRLSIN.cPPT.PGK-GFP.WPRE was carried out through SalI FastD 10 U/µL (Fermentas, Waltham, MA, USA) and AgeI 10 U/µL (Fermentas) in Buffer Orange (Fermentas) for 3 h at 37 °C; the subsequent inactivation was accomplished for 10 min at 65 °C. Then 20 µL of digestion product were dephosphorylated with the Shrimp Alkaline Phosphatase (SAP) 30 min at 37 °C, followed by the enzyme inactivation 10 min at 65 °C. After purification and gel quantification of the digestion products (pRRLSIN.cPPT.PGK.WPRE and HA-rADAR2a/Myc-rADAR2a or HA-rADAR2b/Myc-rADAR2b), the ligation reaction was set up in a volume of 20 µL with a ratio 1:3 of empty vector (50 ng) and HA-rADAR2a/Myc-rADAR2b DNA, respectively, 1 µL T4 DNA Ligase 5 U/µL (Fermentas) and T4 DNA Ligase Buffer 10×; the reaction mixture was maintained 1 h at 22 °C. Once obtained the ligation product, the bacterial transformation was carried out using TOP10 bacteria strain (ThermoFisher).

#### 2.5.2. Lentivirus Particles Production

HEK293T cells were seeded at low passages (no more than P12–15) 24 h before transfection at a density of 9.5 × 10^6^ cells in 150 mm dish; the medium was changed 2 h before transfection. The plasmids mix for one dish was prepared by adding 7 µg VSV-G envelope gene in pMD2.G backbone vector, 16.25 µg PACKAGING plasmids pCMV ΔR8.74 II Gen.Pack, 6.25 µg pRSV-rev plasmid, 32 µg TRANSFER Vector containing the transgene. The TRANFER Vector is the modified pRRLSIN.cPPT.PGK-GFP.WPRE (addgene 12252) in which the GFP sequence has been replaced with HA-rADAR2a/Myc-rADAR2a sequence or HA-rADAR2b/Myc-rADAR2b, as described above.

The plasmid solution was made up with a final volume of 1225 µL with 0.1× Tris EDTA (TE 0.1×)/dH_2_O (2:1); finally, 125 µL of 2.5 M CaCl_2_ were added to the suspension; the mixture was maintained 5 min at RT. The precipitate was formed by adding dropwise 1250 µL of 2× HBS solution to the mixture. The suspension was added immediately to the cells following the addition of 2× HBS. The calcium-phosphate plasmid DNA mixture was allowed to stay on the cells for 14–16 h and thereafter the medium was replaced with fresh medium. After 24 h and 48 h from medium replacement, the cells supernatant was collected. Then, after the ultracentrifugation of the supernatant at 23,000 rpm/2 h at 4 °C, the viral pellet was resuspended in PBS.

### 2.6. HEK293T Cell Cultures Stably Expressing ADAR2a/ADAR2b Enzymes

Stable HEK293T cell lines expressing HA tagged ADAR2a or ADAR2b enzyme, were generated after viral infection at MOI 5 (addgene:12,252). The viral particles were maintained on the cells for 48 h; the medium was changed with fresh medium and the cloning selection of HA positive colonies led to stable cell lines generation. HEK293T cell lines were cultured at 37 °C and 5% CO_2_ in DMEM medium (Invitrogen-ThermoFisher) supplemented with 10% of heat-inactivated fetal bovine serum (FBS), 30 U/mL penicillin (Sigma-Aldrich), 30 µg/mL streptomycin (Sigma-Aldrich), 1% minimum Eagle’s medium nonessential amino acids, 1 mM sodium pyruvate.

#### Primary Neuronal Cultures Viral Transduction

The viral transduction was performed on primary cortical neurons at day in vitro (DIV)9, dropping the viral particles expressing HA-ADAR2a and HA-ADAR2b directly in the medium at MOI 5 leading to a 95–100% transduction efficiency. At DIV14, RNA and proteins were extracted following the procedures reported below.

### 2.7. Transient Transfection

HEK293T cells were plated 24 h before transfection at a density of 30,000 cells/cm^2^; the medium was changed 2 h before transfection. Either 2.5 µg (for 6-well plates) or 0.5 µg (for 24-well plates) of vector [29] were mixed with 0.1× Tris EDTA (TE 0.1×)/dH_2_O (2:1) and 2.5 M CaCl_2_; the mixture was maintained for 5 min at room temperature (RT). The precipitate was formed by adding dropwise 2× HBS solution to the mixture, then the suspension was added immediately to the cells. The calcium-phosphate plasmid DNA mixture was allowed to stay on the cells for 14–16 h, after which the medium was replaced with fresh medium.

### 2.8. In Vitro RNA Editing Assay

For RNA editing assay a vector, expressing Red Fluorescent Protein (RFP) and GFP separated by an editable stop codon (RNAG vector), was used (gift from Prof. Jantsch, Medical University of Vienna). Briefly, the stem-loop containing the R/G editing site of glutamate receptor subunit 2 was modified to contain an amber stop codon at the editing site [29]. The substrate stem-loop was cloned between the RFP and GFP open reading frames (ORFs). The transient transfection of this construct induces the constitutive expression of RFP. The stop codon in the loop prevents GFP translation without editing process; otherwise, the increase of editing levels leads to a conversion of the stop codon to a tryptophan codon inducing the production of GFP. The ratio between the GFP and RFP fluorescence values indicates the editing levels in the cell population: when the editing activity increases, the GFP expression increases as well.

HEK293T cell lines stably expressing ADAR2 isoforms were seeded at a density of 30,000 cells/cm^2^. Twenty-four hours later, the transient transfection with RNAG vector was performed, following the protocol reported in Section 2.7. After 16 h, the samples were collected and maintained in PBS EDTA 2 mM until the FACS analysis was performed.

The samples were read on a MACSQuant flow cytometer (Miltenyi Biotec, Bergisch Gladbach, Germany) and analyzed with FlowJo (Tree Star Inc., Ashland, KY, USA). Editing efficiencies were determined by calculating the ratio of green to red arithmetic mean fluorescence of cells with solid RFP expression as previously described [30].

At least 10,000 events were collected for each sample; the experiment were done in triplicate (30,000 total cells counted) and statistical analysis was performed on the mean value obtained from each experiments (*n* = 3) using one-way analysis of variance (ANOVA) followed by Bonferroni post hoc test.

### 2.9. Quantitative Real Time PCR

For evaluating the mRNA expression of ADAR2, the Real-time PCR was performed and the “Taqman” probes were used through the thermal cycler Applied Biosystem 7500. The RNA expression pattern of the genes of interest was analyzed using Applied Biosystems 7500 real-time PCR system (Life Technologies, Foster City, CA, USA). PCR was carried out using TaqMan Universal PCR Master Mix (Life Technologies), which contained AmpliTaq Gold DNA Polymerase, AmpErase UNG, dNTPs with dUTP, passive reference and optimized buffer components. AmpErase UNG treatment was used to prevent the possible reamplification of carry-over PCR products. Thermal cycling was started by incubation at 50 °C for 2 min and at 95 °C for 10 min for optimal AmpErase UNG activity and activation of AmpliTaq Gold DNA polymerase. After this initial step, 40 cycles of PCR were performed. Each PCR cycle consisted of heating at 95 °C for 15 s for melting and 60 °C for 1 min for annealing and extension. Then, 20 ng of sample were used in each realtime PCR reaction in a final volume of 20 µL. The expression of the target gene ADAR2 (ADAR2: Rn00563671_m1) was calculated using the ddct methods, using the geometric mean of two housekeeping genes (GAPDH: Rn99999916_s1; H2AFZ: Rn00821133_g1).

### 2.10. Protein Extraction, Quantification and Western Blot

Cells harvested from infected primary cortical cultures were solubilized with modified RIPA (50 mM Tris-HCl, pH 7.4, 150 mM NaCl, 1 mM EDTA, 1% IGEPAL CA630, 0.25% NaDOC, 0.1% SDS, 1% NP-40 and Roche (Basel, Switzerland) protease inhibitor tablets) and then sonicated. A portion of the lysate was used for the bicinchoninic acid (BCA) protein concentration assay (Sigma-Aldrich). Equal amounts of protein were applied to precast SDS polyacrylamide gels (4–12% NuPAGE Bis-Tris gels; Invitrogen-ThermoFisher), and the proteins were electrophoretically transferred to a nitrocellulose transfer membrane (GE Healthcare, Waukesha, WI, USA) for 2 h. For detecting endogenous ADAR2, the membrane was incubated for 1 h at RT with 3% non-fat dry milk in TBS-T 0.2%; the primary antibody was used for an overnight incubation at 4 °C (1:350, Abcam (Cambridge, UK) cod: Ab64830). For the GAPDH housekeeping gene, the membrane was incubated for 1 h at RT with 5% non-fat dry milk in TBS-T; the mouse monoclonal anti-GAPDH (1:10,000, Millipore Billerica, MA 01821; cod: MAB374) was used for overnight incubation at 4 °C. For detection, after 3 washes in TBS-T, the membranes were incubated for 1 h at RT with anti-rabbit secondary antibody (IR-Dye, LI-COR Biosciences, Lincoln, NE, USA) cod: 926-32211) or anti-mouse secondary antibody (IR-Dye cod: 926-68020), both diluted 1:2000 in TBS-T. Signals were detected using an Odyssey infrared imaging system (LI-COR Biosciences) and quantified using Odyssey version 1.1 (LI-COR Biosciences).

### 2.11. Proximity Ligation Assay

The Duo-link Proximity Ligation Assay (PLA) Technology kit (Sigma-Aldrich) was used for the proximity ligation assay, accordingly to the manufacturer instructions with minor modifications. Briefly, HEK293T cells were fixed with 4% paraformaldehyde (PFA). Each sample was permeabilized with PBS-Triton 0.3% and then incubated with the blocking solution (Roche) for about 45 min at RT; the primary antibodies incubation was performed overnight at 4 °C with mouse anti c-Myc (Santa Cruz Biotechnology, Dallas, TX, USA; cod: SC40) and rabbit anti-HA (Sigma-Aldrich, cod: H6908). On the following day, the samples were washed three times in Buffer A at RT and then the cells were incubated 1 h at 37 °C with the PLA probe containing the secondary antibodies conjugated with the DNA probes. After PLA probe removal, the samples were washed 4 times × 10 min with the Buffer A at 37 °C. After an additional brief wash with Buffer A at 37 °C, the samples were incubated with the ligation buffer containing oligonucleotides that hybridize to the PLA probe and the DNA ligase which allows the annealing between probe and oligonucleotides to form a rolling circle DNA strand. This reaction was incubated for 30 min at 37 °C. Subsequently, the cells were washed with Buffer A at 37 °C and then incubated with the amplification-detection solution containing the DNA polymerase for rolling circle amplification (100 min at 37 °C). Next, the samples were washed four times with Buffer B at RT and four times with Buffer B 0.01×; then, the coverslips were incubated for 10 min with mounting buffer containing DAPI and analyzed with a confocal microscope.

The dimerization study was performed on a minimum of 50 cells for each type of transfection (ADAR2b-Myc and ADAR2b-HA or ADAR2a-Myc and ADAR2a-HA or ADAR2b-Myc and ADAR2a-HA); the PLA spots were automatically counted with ImageJ 1.46 r and the mean number of spot/cell was calculated for each sample. Untransfected HEK293T cells were used as negative control.

### 2.12. Homology Modelling of ADAR2 Splicing Isoforms and Molecular Dynamics

To find suitable template for homology modeling, we used the protein sequence of ADAR2b (NP_037026.2), the longest isoform, as query in protein Basic Local Alignment Search Tool BLAST search against Protein Data Bank (PDB) database. We selected 5HP2 [31] structure as optimal template, given high sequence identity (93%) with ADAR2b. This structure also includes an RNA chain bounded to the enzyme, which allows evaluation of protein-RNA interaction in our prediction model. For each rat isoform, ADAR2a (NP_001104525.1) and ADAR2b (NP_037026.2), 3D structure was predicted by homology modeling based on the 5HP2 template using I-Tasser [32] with default parameters. Based on analysis of local structure quality, predicted structures were restrained to the C-terminal deaminase domains (from amino acid 305), that were predicted with higher confidence scores (Supplementary Figure S3A,B). Predicted structures were then superimposed on the 5HP2 template to assess the position of RNA chain and obtain a prediction of the whole protein-RNA complexes. Inositol hexakisphosphate (IHP), which is present in the template structure, was not included in our models. Each isoform was studied both with and without RNA. The original structure of RNA was modified by substitution of 8-azanebularine with a standard adenosine. A zinc ion is also present in the crystal structure; simulations were performed both with and without this ion. Molecular dynamics (MD) simulations for each model were performed by means of GROMACS 5.0.4 [33,34] with the amber99-sb force field, which also includes parameters for nucleic acids. The molecule was solvated in an octahedral box, with a 1 nm minimum distance between the solute and the box boundary. The electric charge of the system was neutralized and a 150 mM salt concentration was simulated by addition of a suitable number of Na^+^ and Cl^−^ ions. Coulomb interactions were treated by the PME (particle-mesh Ewald) method, while van der Waals interactions were calculated within a cutoff of 1.0 nm. Temperature control was ensured by a velocity rescaling algorithm with a time constant of 0.1 ps. Pressure control was based on the Parrinello–Rahman algorithm with a time constant of 1 ps and compressibility of 4.5 × 10^−5^ bar^−1^. All simulations were run at 300 K and 1 atm. In each simulation, the energy of the system was first minimized by steepest descent. Then, two 100 ps equilibration phases followed, with position restraints on the solute, the first at constant volume (NVT), with initial velocities of atoms randomly assigned according to a Maxwell distribution, and the second at constant pressure (NPT). After equilibration, restraints were removed and a 30 ns NPT simulation was carried out. Four such simulations were executed with different initial velocities: the last 20 ns of the resulting trajectories were then concatenated, giving one 80 ns trajectory, which was used for subsequent analysis. Root mean square fluctuation (RMSF) and hydrogen bond analyses were executed by means of the utilities available in the GROMACS package. For the calculation of overlapping volume, a computer program was developed. Graphical representations of structures were generated by means of visual molecular dynamics VMD [35].

### 2.13. Statistical Analysis

All experiments were performed on at least three independent cellular cultures, both for HEK293T cells and primary cortical neurons. Results of Western blot (WB), qPCR, Agilent quantification, editing level quantification are represented as the mean and standard error of the mean (SEM) of the three biological replicates for each experimental point. Statistical analysis was performed with one-way ANOVA, followed by Dunnett post-hoc test or Bonferroni post-hoc test.

## 3. Results

### 3.1. ADAR2 Expression Increase during Primary Cortical Neuron Culture Maturation

**Figure 2:**
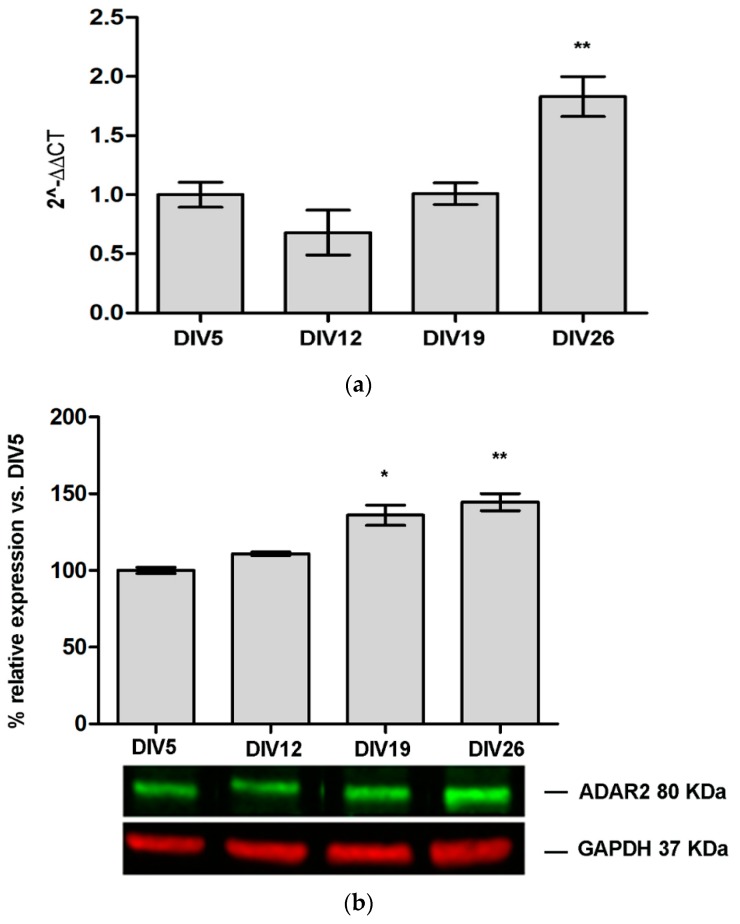
Rat ADAR2 expression during cortical neuron cell cultures maturation. The graphs indicate the increase compared to day in vitro (DIV) 5: (**a**) mRNA expression level of rat ADAR2; and (**b**) Western blot analysis for rat ADAR2 protein expression. Data are represented as means ± standard error of the mean (SEM) (*n* = 3). Statistical analysis was performed using one-way ANOVA followed by Dunnett post hoc test (* *p* value < 0.05; ** *p* value < 0.01, vs. DIV5).

To have a complete picture of ADAR2 expression during primary cortical neurons maturation (Figures S1 and S2), RT-PCR and WB analysis were performed at several time points (DIV5, DIV12, DIV19, and DIV26). As indicated in Figure 2a, ADAR2 mRNA is stable until DIV19 and then it strongly increases at DIV26 (DIV26 1.83 ± 0.17, *p* value < 0.01 vs. CTR DIV5). WB analysis (Figure 2b) showed that ADAR2 protein is subjected to a progressive increase of 40–45% at DIV19 and DIV26, respectively, compared to DIV5.

### 3.2. ADAR2 Self-Editing and Splicing Pattern Is Modified during Cortical Neurons Development

**Figure 3:**
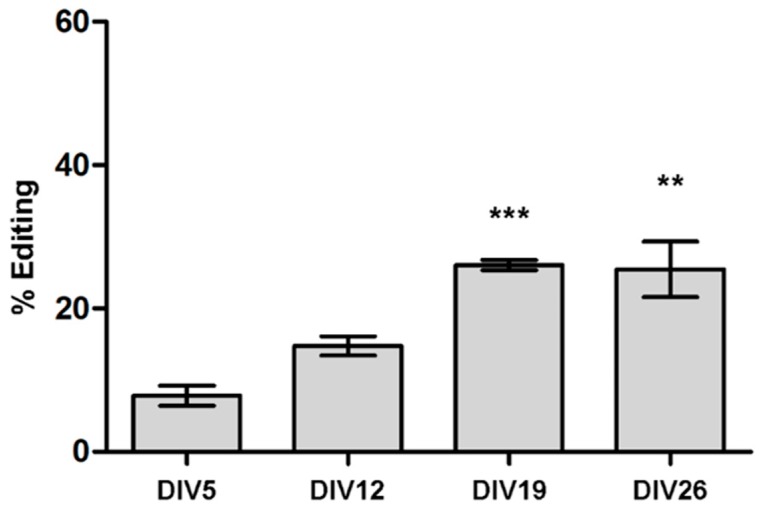
RNA editing levels for ADAR2 in the −1 self-editing site during cortical neuron cell cultures maturation compared to DIV5. Data are represented as means ± SEM (*n* = 3). Statistical analysis was performed using one-way ANOVA followed by Dunnett post hoc test (** *p* value < 0.01; *** *p* value < 0.001 vs. DIV5).

To understand ADAR2 enzymatic regulations, we monitored the self-editing process on ADAR2 transcript itself and its splicing mechanisms, during primary cortical neurons maturation at several time points. A progressive increase of self-editing levels, reaching a maximum at DIV19 was found. Specifically, at DIV5, 8 ± 1.4% of edited transcripts were detected; at DIV12, 14 ± 1.3% (not significant (n.s.)); at DIV19 and DIV26, editing levels reached 26 ± 0.72%, *p* value < 0.001, and 25.5 ± 3.84%, *p* value < 0.01, vs. DIV5 respectively (Figure 3).

**Figure 4:**
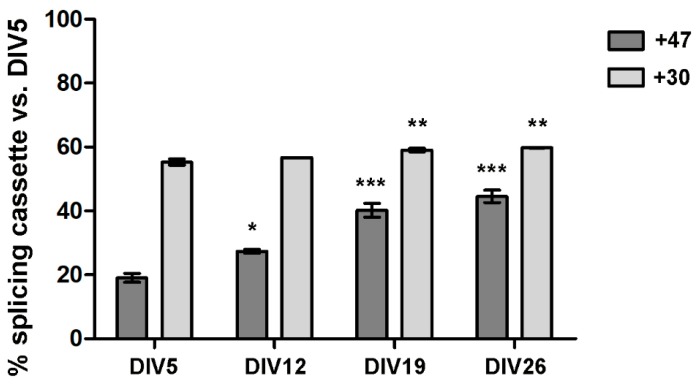
Inclusion of the 47 nt and 30 nt cassettes in ADAR2 transcript during cortical neuron cell cultures maturation; reported data are compared to DIV5. Data are represented as means ± SEM (*n* = 3). Statistical analysis was performed using one-way ANOVA followed by Dunnett post hoc test (* *p* value < 0.05; ** *p* value < 0.01; *** *p* value < 0.001 vs. DIV5).

Since self-editing leads to the inclusion of a 47 nt cassette generating a splicing variant that encodes for a truncated protein without editing activity, we tested the expression of this splicing cassette. During the maturation process, a progressive increase of this cassette inclusion can be observed in ADAR2 transcripts, starting from values of 19 ± 1.37% at DIV5 and reaching levels of 27.4 ± 0.59%, *p* value < 0.05, vs. DIV5, 40.2 ± 2.18%, *p* value < 0.001, and 44.6 ± 1.95%, *p* value < 0.001, at DIV12, -19 and -26 vs. DIV5 respectively (Figure 4).

Next, we focused on the splicing event that modifies the mRNA sequence coding for the catalytic domain with the insertion of a 30 nt cassette, leading to the so-called ADAR2a and ADAR2b splicing isoforms. The transcripts containing this splicing cassette represent 55.3 ± 0.97% of the transcripts at DIV5, with slightly higher values in subsequent time points (56.7 ± 0.07% n.s. vs. DIV5 at DIV12, 59.08 ± 0.57% at DIV19, *p* value < 0.01, vs. DIV5, 59.8 ± 0.07, *p* value < 0.01, at DIV26 vs. DIV5) (Figure 4).

### 3.3. ADAR2a Splicing Isoform Has the Higher Enzymatic Activity

**Figure 5:**
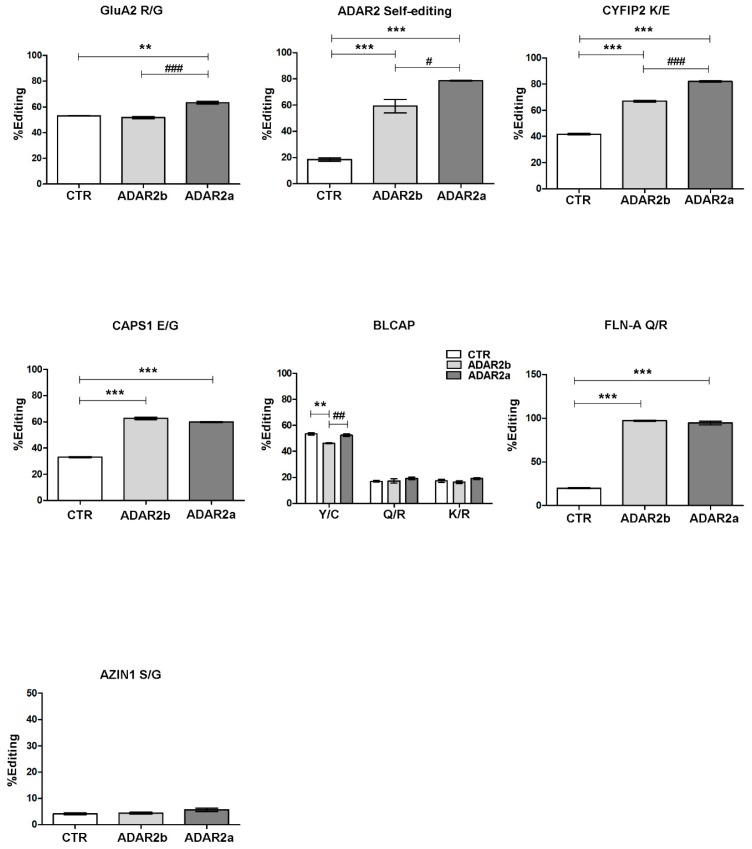
RNA editing levels in DIV14 cortical neurons overexpressing ADAR2 splicing isoforms after lentiviral infection. White bar: not infected neurons. Gray bar: ADAR2b infected neurons; Dark gray bar: ADAR2a infected neurons. Data are presented as means ± SEM (*n* = 3). Statistical analysis was performed using one-way ANOVA followed by Bonferroni post hoc test (** *p* value < 0.01; *** *p* value < 0.001 vs. CTR; ^#^ *p* value < 0.05; ^##^ *p* value < 0.01; ^###^ *p* value < 0.001 vs. ADAR2b).

Since both ADAR2 isoforms are almost equally expressed during neuronal maturation and the RNA editing levels generally increase with development [26,36], the RNA editing activity of these isoforms was investigated. RNA editing levels for several transcripts involved in Central Nervous System (CNS) physiology were evaluated in primary cortical neurons infected with lentivirus particles carrying ADAR2a or ADAR2b expression vectors (Figure 5; Figure S3 shows that the two isoforms are equally expressed).

As expected, we found a statistically significant increase in editing levels in almost all the sites analyzed after overexpression of both ADAR2 isoforms. Interestingly, increased editing activity compared to ADAR2b was observed for ADAR2a on specific editing sites, i.e., GluA2 R/G site (ADAR2b: 51.8 ± 0.97%; ADAR2a: 63.3 ± 1.06% *p* value < 0.001 vs. ADAR2b), ADAR2 self-editing site (ADAR2b: 59.3 ± 5.13%; ADAR2a: 78.6 ± 0.2%, *p* value < 0.05 vs. ADAR2b), and CYFIP2 K/E site (ADAR2b: 67 ± 0.67%; ADAR2a: 82 ± 0.5% *p* value < 0.001 vs. ADAR2b).

Furthermore, overexpression of ADAR2 isoforms affects only Y/C site of BLCAP transcript, but with peculiar effects: ADAR2b decreased the editing level (CTR: 53.4 ± 0.88%; ADAR2b: 46.3 ± 0.31% *p* value < 0.01 vs. CTR), whereas ADAR2a seemed to have no significant effect compared to control sample. Eventually, no statistically significant differences in editing levels were reported in AZIN1 transcript, an ADAR1 specific substrate.

Evaluating the 5-HT_2c_-R editing site, as shown in Table 1, almost all sites underwent a modulation in their levels after ADAR2 infection. In particular, a significant decrease in editing levels was seen for site A and B (ADAR1 specific sites [37]) compared to control samples; for the B site, ADAR2a isoform induced a further downregulation compared to ADAR2b. A different picture was observed for sites C′, C and D, since an increase in RNA editing levels was found.

All sets of analysis were also performed in parallel on PC12 cell lines physiologically expressing the transcripts of interest and transduced with lentiviral particles carrying ADAR2a or ADAR2b splicing isoforms. The results obtained were comparable with those reported above (Figures S4 and S5).

Furthermore, we set up an in vitro editing assay [29] in HEK293T cells stably expressing ADAR2a or ADAR2b enzyme in combination with an editable target. The target was generated by a vector (RNAG) expressing RFP and GFP proteins separated by an editable stop codon within GluA2 R/G stem loop [29]. Upon editing, the stop codon was converted to a tryptophan codon, allowing the expression of GFP. The ratio between GFP and RFP fluorescence values indicates the level of editing.

**Figure 6:**
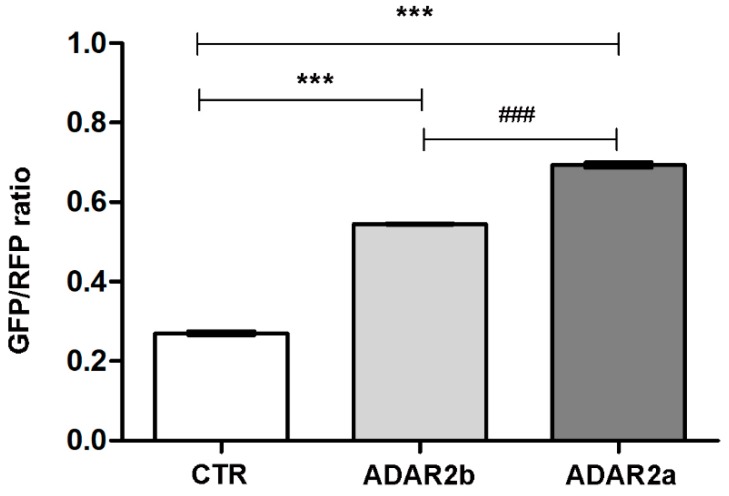
Editing Assay in HEK293T cells. Increase of the green to red fluorescence ratio indicates an increase in editing level. White bar: HEK293T cells transfected with RNAG vector alone; Gray bar: HEK293T cells stably expressing ADAR2b and transfected with RNAG; Dark gray bar: HEK293T cells stably expressing ADAR2a and transfected with RNAG. Data are presented as means ± SEM (*n* = 3). Statistical analysis was performed using one-way ANOVA followed by Bonferroni post hoc test (*** *p* value < 0.001 vs. CTR; ^###^ *p* value < 0.001 vs. ADAR2b). GFP: Green fluorescent protein; RFP: Red fluorescent protein.

HEK293T cells transiently transfected only with the construct RNAG showed a GFP/RFP ratio of 0.270 ± 0.004 and this is consistent with the low enzymatic activity previously observed in this cell line [38]. HEK293T cells stably expressing ADAR2b and transiently expressing the RNAG reporter were found to have a GFP/RFP ratio of 0.545 ± 0.001 (*p* value < 0.001 vs. CTR); HEK293T stably over-expressing ADAR2a and transiently expressing the RNAG reporter showed an increased GFP/RFP ratio to 0.693 ± 0.007 (*p* value < 0.001 vs. CTR, *p* value < 0.001 vs. HEK293T over-expressing ADAR2b) (Figure 6). This result supports our previous evidence, confirming the ADAR2a as the most active splicing isoform.

### 3.4. ADAR2 Splicing Isoforms Do Not Modify the Enzyme Dimerization Properties

**Figure 7:**
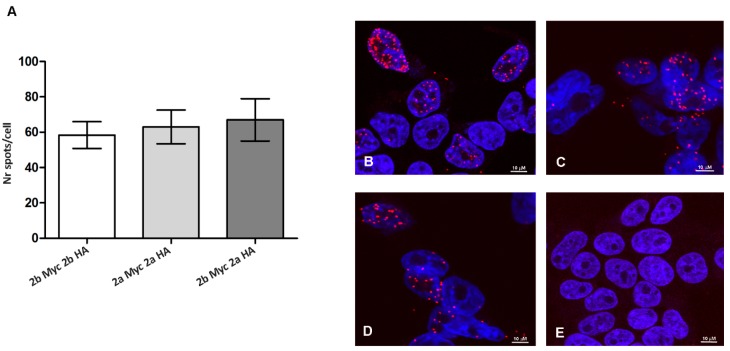
PLA detection of ADAR2 dimers formation in HEK293T transfected cells. (**A**) PLA spots quantification. The graph reports the mean number of spots/cell; a minimum of 50 cells were analyzed for each experimental point; statistical analysis was performed using one-way ANOVA followed by Bonferroni post hoc test; (**B**) PLA dots for ADAR2b-Myc and ADAR2b-HA interaction; (**C**) PLA dots for ADAR2a-Myc and ADAR2a-HA interaction; (**D**) PLA dots for ADAR2b-Myc and ADAR2a-HA interaction; and (**E**) negative control: HEK293T Untransfected cells. Scale bar 10 µM.

Since we obtained evidence of a different activity for ADAR2 splicing isoforms, we wondered if this could be due to a different homodimerization between the splicing isoforms themselves. We performed PLA experiment after transient transfection of HEK293T cells with different combination of tagged ADAR2 splicing isoforms (Figure 7). As reported in Figure 7A, the mean number of PLA spots in each cell showed no statistical significant difference in the samples analyzed (ADAR2b-Myc and ADAR2b-HA 58 ± 3.72; ADAR2a-Myc and ADAR2a-HA 63 ± 4.01; ADAR2b-Myc and ADAR2a-HA 63 ± 8.43), therefore showing no modulation in proteins dimerization capability. Representative images of each sample are reported in Figure 7B–E.

### 3.5. Molecular Dynamics Suggests Different Interactions with RNA Chain for ADAR2 Isoforms

The computational techniques of homology modeling and MD were applied to investigate the structural properties that can affect the interactions of ADAR2 with RNA. For each isoform a model was built by homology modeling based on the crystal structure of human ADAR2 (5HP2) using I-Tasser: rat ADAR2a (NP_001104525.1) with root mean square deviation (RMSD) 18.5 ± 2.2 and rat ADAR2b (NP_037026.2) with RMSD 18.5 ± 2.2.

For subsequent analysis, predicted structures were restrained to the C-terminal region (from amino acid Pro 305), that were predicted with higher confidence scores and correspond to the deaminase domain complexed with RNA, defined in the template crystal structure 5HP2 (Figure S6A,B). In this way, we assured to perform the MD study using a confident predicted model.

**Figure 8:**
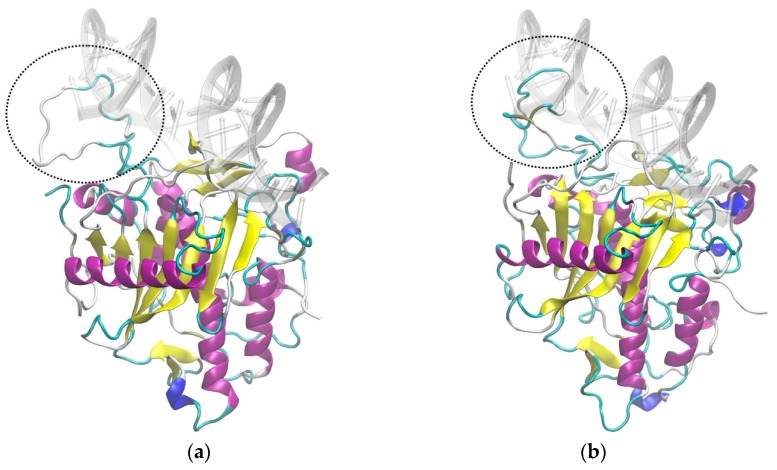
Structures of: ADAR2a (**a**); and ADAR2b (**b**) from MD simulations. The RNA shown is taken from the PDB crystal structure 5HP2 after fitting of the protein backbone atoms. The two selected structures are those with maximum overlap with RNA among those collected in the respective MD simulations. Dashed circles show the main overlap region.

In particular, the sequence of ADAR2b contains ten additional amino acids with respect to ADAR2a, after residue 465, in a loop located very close to the RNA binding site (Figure 8). Starting from the structure thus determined for each model, MD simulations were performed for the protein alone and for the protein bound to RNA, without any restraint on the RNA structure, both including and excluding the Zn^++^ ion, which is reported in the original PDB structure of human ADAR2.

**Figure 9:**
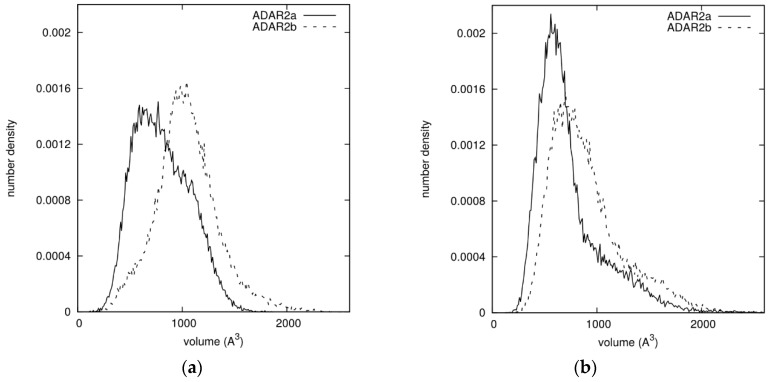
Volume overlap between ADAR2 atoms in the MD simulations of the protein alone, and RNA atoms in the model structure. The results for ADAR2a (continuous line) are compared with those for ADAR2b (dashed line): (**a**) MD simulations without Zn^++^ ion; and (**b**) MD simulations with Zn^++^ ion.

For both isoforms the mobility of ADAR2 residues was analyzed in the presence and in the absence of RNA, by calculating the RMSF of each residue, as shown in Figure S7. The highest values of RMSF, excluding the tails, were found between residues 450 and 500, approximately corresponding to the loop containing the extra-residues of ADAR2b, and such values were modified by the presence of RNA. The addition of the Zn^++^ ion slightly alters some local properties, but the highest fluctuations remain located in the same loop and the effect of RNA is still significant. These results suggest that the loop could interfere with the approach of RNA to the binding site. The superposition of ADAR2 atoms with the region potentially occupied by RNA was then analyzed as follows: each structure collected during the simulations of the protein alone was superimposed to the initial model structure with RNA, and the overlapping volumes of all protein atoms with RNA atoms were calculated; the sum of all such values was taken as overlapping volume between the protein and the space to be occupied by RNA in case of binding. The resulting density distribution of this quantity is shown in Figure 9 for both isoforms: the distribution for ADAR2b is shifted towards higher values with respect to the one for ADAR2a. The difference between the two isoforms is more evident when the Zn^++^ ion is absent. These simulations strengthen the hypothesis that the additional 10 aa loop might interfere with the binding of RNA.

The different editing activity of ADAR2a and ADAR2b could also depend on differences in some key local interactions with RNA. The time-averaged number of hydrogen bonds between selected residues and RNA was calculated from the MD simulations of the two isoforms, as shown in Table 2. For residues from 351 to 455 the values of ADAR2b are slightly lower than those of ADAR2a, while for residues from 473 to 510 (483 to 520 in ADAR2b) the values of ADAR2b are higher than those of ADAR2a. This indicates a small structural rearrangement of the binding interface, but the results do not suggest a loss of key interactions in the case of ADAR2b with respect to ADAR2a, nor a substantial structural change of RNA. Further details on the presence of hydrogen bonds at each site as a function of time are given in Figure S8. No significant hydrogen bond was found between the ten additional amino acids of ADAR2b and RNA.

## 4. Discussion

RNA editing increases in the developing mouse brain, but the protein levels of the two enzymes are somehow constant and do not seem to follow the increased editing level [15,36,39,40]. To assess the molecular mechanisms that regulate ADAR expression and activity we focus on neuronal maturation of isolated primary cortical neuronal cells, that are frequently used to mimic many features of neuronal development [41,42,43], emphasizing in particular the role of ADAR2 and its splicing isoforms.

We found that ADAR2 expression increases during neuronal maturation both at the mRNA and protein levels as recently reported [15] in a similar neuronal system. However, the slight increase in protein expression does not seem to recapitulate the robust augmentation in the editing level of several sites as reported for both neuronal maturation in vitro [15,26] and brain development in vivo [36,40,44].

Furthermore, the extent of editing varies within different tissues during human embryogenesis and, again, does not always correlate with ADAR expression [44]. To address this feature, several studies focused on ADAR post-translational modifications/cofactors interaction [5,29,45,46,47,48,49,50] and on splicing isoforms activity [19,21]. In particular, the activity of ADAR2 has been shown to be regulated by pre-mRNA processing events including self-editing and several alternative splicing events [19,21,22]. Here, we attempt to elucidate the role of these processes during neuronal maturation. The self-editing process creates an alternative 3′ acceptor splicing site in ADAR2 pre-mRNA; 47 nucleotides are inserted in the coding sequence, giving rise to a premature stop codon leading to a truncated, inactive form of ADAR2 [19,23]. We showed that in rat CNS the self-editing process increased during primary cortical neuron maturation with a parallel augmented expression of the so-called ADAR2e/f isoforms containing the +47 isoform cassette. This is in line with the results shown by Hang et al., (2008) [51], demonstrating that ADAR2 self-editing had already occurred in pre-mRNA during early development of primary cortical neurons, with a progressive increase of editing levels during maturation. A similar result has been recently reported by Behm et al., 2017 [15]. The increased self-editing in the ADAR2 pre-mRNA should lead to the generation of inactive enzyme that seems to be in contrast with the increased editing levels usually reported during development. However, fine tuning ADAR2 activity is necessary for the correct development of neuronal cells. The increase of the inactive ADAR2 isoform does not overwhelm ADAR2 activity at all, but, on the contrary, it might be necessary to avoid an excessive and inappropriate RNA editing throughout the CNS.

The editing activity of ADAR2 is also finely regulated through the splicing event involving the sequence coding for the catalytic domain, leading to the generation of the so-called ADAR2a and ADAR2b isoforms. This splicing event includes 30 nucleotides in mouse and rat ADAR2 (120 nt in human). Since the role of the ADAR2a and b variants has not been fully elucidated and contrasting results among species are reported [19,21], we tried to unveil differences in their expression and enzymatic activity.

The analysis of the expression pattern for the two isoforms during in vitro neuronal maturation, indicated that ADAR2a and ADAR2b mRNA are almost equally expressed with a slightly increase of ADAR2b at DIV19 and DIV26. These data indicate that both splicing isoforms are needed for proper neuronal functions. Unfortunately, no data are reported in the literature showing the relative expression of the two isoforms in adult brain or during brain development, making difficult to determine their biological role in vivo.

Furthermore, ADAR2a seems to be the most active enzyme on GluA2 R/G site, on ADAR2 self-editing site and on CYFIP2 K/E site and on 5-HT_2c_-R C′, C and D sites. These data were confirmed also in our editing assay, using the double stranded loop of GluA2 R/G as target site. However, no difference between the editing activities of the two isoforms was shown for CAPS1 E/G and FLNA-Q/R sites. These data indicate that ADAR2a is generally more active that ADAR2b, but this difference is site and probably structure dependent. The reason in several sites there is not a clear difference between the two isoforms needs further investigations. Interestingly, the editing sites that are specifically or preferentially targeted by ADAR1, such as AZIN1 S/G site, 5-HT_2c_-R A and B sites and BLCAP site, were not affected by ADAR2 overexpression or, surprisingly, they were downregulated. This might indicate that ADAR2 overexpression inhibited ADAR1 activity, without affecting its expression (Figure S9), perhaps through a process of enzyme sequestration, already reported for ADAR1 overexpression towards ADAR2 [5,52].

We wondered, then, if the differential activity between ADAR2a and ADAR2b could be linked to a differential dimerization between isoforms due to the addition of 10 amino acids in ADAR2b. We demonstrated through PLA experiments that all the possible ADAR2 dimer combinations can be formed (A2a/A2a, A2b/A2b, and A2a/A2b) without any statistically significant variation. These results indicate that the 10 amino acids cassette present in ADAR2b does not interfere with the dimerization properties. We went further analyzing if the additional cassette of ADAR2b might have an influence on the properties of ADAR2 structure. The computational techniques of homology modeling and MD were applied to investigate the structural properties that can affect the interactions of ADAR2 splicing isoforms with RNA. Starting from the deposited human ADAR2 structure, we modelled the two rat ADAR2 splicing isoforms with or without the 10 amino acids. Molecular dynamic approaches suggest differential interactions with RNA of the two isoforms. In particular, the 10 amino acids loop of ADAR2b might interfere with RNA binding, occupying the space volume in which the RNA should be present in case of binding. These data indicate that ADAR2b might have a lower capability of binding RNA than ADAR2a and this feature could explain its lower editing efficiency. However, further investigations are needed to conclusively support this hypothesis.

In summary, our data shed light on the regulation of ADAR2 expression and activity. Both ADAR2a and ADAR2b splicing variants are equally expressed during neuronal differentiation. ADAR2b is the less active form due to the insertion of a 10 amino acids loop in the deaminase domain that might interfere with RNA interaction. Our results confirm what found previously for human RED1, in which an Alu-like cassette, located in the homologous position of the rat 30 nt cassette, when spliced in the mRNA generates a less active RED1 isoform. This conserved mechanism of splicing adds complexity to ADAR2 regulations and should be considered when studying ADAR2 activity in neuronal cells.

## Figures and Tables

**Table 1:** 5-HT_2c_R editing levels in DIV14 cortical neurons overexpressing ADAR2 splicing isoforms. Statistical analysis was performed using one-way ANOVA followed by Bonferroni post hoc test (* *p* value < 0.05; ** *p* value < 0.01; *** *p* value < 0.001 vs. CTR; ^###^ *p* value < 0.001 vs. ADAR2b).

5-HT_2c_R Editing			
	CTR	ADAR2b	ADAR2a
**Site A**	79.2 ± 3.83%	54 ± 0.77% **	45.6 ± 1.82% ***
**Site B**	88.7 ± 0.13%	65.2 ± 0.45% ***	57.8 ± 0.59% ***/^###^
**Site C′**	10.9 ± 0.93%	17.6 ± 1.11%	20.6 ± 2.38% *
**Site C**	17 ± 0.46%	23.4 ± 2.38% *	26.2 ± 0.49% **
**Site D**	71.8 ± 0.3%	96.3 ± 0.93% ***	99.1 ± 0.6% ***

**Table 2:** Time averaged number of hydrogen bonds of selected residues with RNA in MD simulations; without (**A**); and with Zn^++^ ion (**B**). Residue number in parentheses refers to ADAR2b.

**A**					
**Residue**	**ADAR2a**	**ADAR2b**	**Residue**	**ADAR2a**	**ADAR2b**
Val 351	0.00	0.00	Lys 475 (485)	0.20	0.69
Thr 375	0.58	0.40	Arg 477 (487)	0.06	1.65
Lys 376	1.20	0.79	Arg 481 (491)	1.85	1.79
Glu 396	0.00	0.00	Ser 486 (496)	1.07	1.28
Cys 451	0.00	0.00	Glu 488 (498)	1.91	2.17
Arg 455	1.30	1.26	Thr 490 (500)	0.00	0.11
Asn 473 (483)	0.23	0.61	Ser 495 (505)	0.00	0.35
Arg 474 (484)	1.09	2.44	Arg 510 (520)	0.69	1.36
**B**					
**Residue**	**ADAR2a**	**ADAR2b**	**Residue**	**ADAR2a**	**ADAR2b**
Val 351	0.00	0.00	Lys 475 (485)	0.37	0.87
Thr 375	0.25	0.30	Arg 477 (487)	0.01	1.27
Lys 376	0.81	0.96	Arg 481 (491)	1.28	0.97
Glu 396	0.00	0.00	Ser 486 (496)	1.12	1.17
Cys 451	0.09	0.00	Glu 488 (498)	1.30	1.73
Arg 455	0.14	0.74	Thr 490 (500)	0.03	0.12
Asn 473 (483)	0.42	0.62	Ser 495 (505)	0.00	0.28
Arg 474 (484)	2.18	1.88	Arg 510 (520)	0.60	0.89

## Supplementary Materials

The following are available online at http://www.mdpi.com/2073-4425/9/2/79/s1.

## References

[1] Tariq A., Jantsch M.F. (2012). Transcript diversification in the nervous system: A to I RNA editing in CNS function and disease development. Front. Neurosci..

[2] Keegan L.P., Gallo A., O’Connell M.A. (2001). The many roles of an RNA editor. Nat. Rev. Genet..

[3] Maas S., Rich A., Nishikura K. (2003). A-to-I RNA editing: Recent news and residual mysteries. J. Biol. Chem..

[4] Bazak L., Haviv A., Barak M., Jacob-Hirsch J., Deng P., Zhang R., Isaacs F.J., Rechavi G., Li J.B., Eisenberg E. (2014). A-to-I RNA editing occurs at over a hundred million genomic sites, located in a majority of human genes. Genome Res..

[5] Chilibeck K.A., Wu T., Liang C., Schellenberg M.J., Gesner E.M., Lynch J.M., MacMillan A.M. (2006). FRET analysis of in vivo dimerization by RNA-editing enzymes. J. Biol. Chem..

[6] Song C., Sakurai M., Shiromoto Y., Nishikura K. (2016). Functions of the RNA editing enzyme ADAR1 and their relevance to human diseases. Genes.

[7] Peng P.L., Zhong X., Tu W., Soundarapandian M.M., Molner P., Zhu D., Lau L., Liu S., Liu F., Lu Y. (2006). ADAR2-dependent RNA editing of AMPA receptor subunit GluR2 determines vulnerability of neurons in forebrain ischemia. Neuron.

[8] Yamashita T., Hideyama T., Hachiga K., Teramoto S., Takano J., Iwata N., Saido T.C., Kwak S. (2012). A role for calpain-dependent cleavage of TDP-43 in amyotrophic lateral sclerosis pathology. Nat. Commun..

[9] Barbon A., Fumagalli F., Caracciolo L., Madaschi L., Lesma E., Mora C., Carelli S., Slotkin T.A., Racagni G., Di Giulio A.M. (2010). Acute spinal cord injury persistently reduces R/G RNA editing of AMPA receptors. J. Neurochem..

[10] Patterson J.B., Samuel C.E. (1995). Expression and regulation by interferon of a double-stranded-RNA-specific adenosine deaminase from human cells: Evidence for two forms of the deaminase. Mol. Cell. Biol..

[11] Strehblow A., Hallegger M., Jantsch M.F. (2002). Nucleocytoplasmic distribution of human RNA-editing enzyme ADAR1 is modulated by double-stranded RNA-binding domains, a leucine-rich export signal, and a putative dimerization domain. Mol. Biol. Cell.

[12] Maas S., Gommans W.M. (2009). Identification of a selective nuclear import signal in adenosine deaminases acting on RNA. Nucleic Acids Res..

[13] Desterro J.M., Keegan L.P., Lafarga M., Berciano M.T., O’Connell M., Carmo-Fonseca M. (2003). Dynamic association of RNA-editing enzymes with the nucleolus. J. Cell Sci..

[14] Sansam C.L., Wells K.S., Emeson R.B. (2003). Modulation of RNA editing by functional nucleolar sequestration of ADAR2. Proc. Natl. Acad. Sci. USA.

[15] Behm M., Wahlstedt H., Widmark A., Eriksson M., Ohman M. (2017). Accumulation of nuclear ADAR2 regulates adenosine-to-inosine RNA editing during neuronal development. J. Cell Sci..

[16] Orlandi C., Barbon A., Barlati S. (2012). Activity regulation of adenosine deaminases acting on RNA (ADARs). Mol. Neurobiol..

[17] Oakes E., Anderson A., Cohen-Gadol A., Hundley H.A. (2017). Adenosine Deaminase That Acts on RNA 3 (ADAR3) Binding to glutamate receptor subunit B pre-mRNA inhibits RNA editing in glioblastoma. J. Biol. Chem..

[18] Chen C.X., Cho D.S., Wang Q., Lai F., Carter K.C., Nishikura K. (2000). A third member of the RNA-specific adenosine deaminase gene family, ADAR3, contains both single- and double-stranded RNA binding domains. RNA.

[19] Rueter S.M., Dawson T.R., Emeson R.B. (1999). Regulation of alternative splicing by RNA editing. Nature.

[20] Feng Y., Sansam C.L., Singh M., Emeson R.B. (2006). Altered RNA editing in mice lacking ADAR2 autoregulation. Mol. Cell. Biol..

[21] Gerber A., O’Connell M.A., Keller W. (1997). Two forms of human double-stranded RNA-specific editase 1 (hRED1) generated by the insertion of an Alu cassette. RNA.

[22] Lai F., Chen C.X., Carter K.C., Nishikura K. (1997). Editing of glutamate receptor B subunit ion channel RNAs by four alternatively spliced DRADA2 double-stranded RNA adenosine deaminases. Mol. Cell. Biol..

[23] Slavov D., Gardiner K. (2002). Phylogenetic comparison of the pre-mRNA adenosine deaminase ADAR2 genes and transcripts: Conservation and diversity in editing site sequence and alternative splicing patterns. Gene.

[24] Kawahara Y., Ito K., Ito M., Tsuji S., Kwak S. (2005). Novel splice variants of human ADAR2 mRNA: Skipping of the exon encoding the dsRNA-binding domains, and multiple C-terminal splice sites. Gene.

[25] Maas S., Gommans W.M. (2009). Novel exon of mammalian ADAR2 extends open rReading frame. PLoS ONE.

[26] Orlandi C., La Via L., Bonini D., Mora C., Russo I., Barbon A., Barlati S. (2011). AMPA receptor regulation at the mRNA and protein level in rat primary cortical cultures. PLoS ONE.

[27] Barbon A., Vallini I., La Via L., Marchina E., Barlati S. (2003). Glutamate receptor RNA editing: a molecular analysis of GLUluR2, GLUR5 and GLUR6 in human brain tissues and in NT2 cells following in vitro neural differentiation. Mol. Brain Res..

[28] La Via L., Bonini D., Russo I., Orlandi C., Barlati S., Barbon A. (2013). Modulation of dendritic AMPA receptor mRNA trafficking by RNA splicing and editing. Nucleic Acids Res..

[29] Garncarz W., Tariq A., Handl C., Pusch O., Jantsch M.F. (2013). A high-throughput screen to identify enhancers of ADAR-mediated RNA-editing. RNA Biol..

[30] Schoft V.K., Schopoff S., Jantsch M.F. (2007). Regulation of glutamate receptor B pre-mRNA splicing by RNA editing. Nucleic Acids Res..

[31] Matthews M.M., Thomas J.M., Zheng Y., Tran K., Phelps K.J., Scott A.I., Havel J., Fisher A.J., Beal P.A. (2016). Structures of human ADAR2 bound to dsRNA reveal base-flipping mechanism and basis for site selectivity. Nat. Struct. Mol. Biol..

[32] Yang J., Yan R., Roy A., Xu D., Poisson J., Zhang Y. (2015). The I-TASSER suite: Protein structure and function prediction. Nat. Methods.

[33] Hess B., Kutzner C., van der Spoel D., Lindahl E. (2008). GROMACS 4: Algorithms for highly efficient, load-balanced, and scalable molecular simulation. J. Chem. Theory Comput..

[34] Abraham M.J., Murtola T., Schulz R., Pall S., Smith J.C., Hess B., Lindahl E. (2015). GROMACS: High performance molecular simulations through multi-level parallelism from laptops to supercomputers. SoftwareX.

[35] Humphrey W., Dalke A., Schulten K. (1996). VMD: Visual molecular dynamics. J. Mol. Graph..

[36] Hwang T., Park C.K., Leung A.K., Gao Y., Hyde T.M., Kleinman J.E., Rajpurohit A., Tao R., Shin J.H., Weinberger D.R. (2016). Dynamic regulation of RNA editing in human brain development and disease. Nat. Neurosci..

[37] Hartner J.C., Schmittwolf C., Kispert A., Muller A.M., Higuchi M., Seeburg P.H. (2004). Liver disintegration in the mouse embryo caused by deficiency in the RNA-editing enzyme ADAR1. J. Biol. Chem..

[38] Melcher T., Maas S., Herb A., Sprengel R., Seeburg P.H., Higuchi M. (1996). A mammalian RNA editing enzyme. Nature.

[39] Hideyama T., Teramoto S., Hachiga K., Yamashita T., Kwak S. (2012). Co-occurrence of TDP-43 mislocalization with reduced activity of an RNA editing enzyme, ADAR2, in aged mouse motor neurons. PLoS ONE.

[40] Ekdahl Y., Farahani H.S., Behm M., Lagergren J., Ohman M. (2012). A-to-I editing of microRNAs in the mammalian brain increases during development. Genome Res..

[41] Lesuisse C., Martin L.J. (2002). Long-term culture of mouse cortical neurons as a model for neuronal development, aging, and death. J. Neurobiol..

[42] Walsh K., Megyesi J., Hammond R. (2005). Human central nervous system tissue culture: A historical review and examination of recent advances. Neurobiol. Dis..

[43] Baj G., Patrizio A., Montalbano A., Sciancalepore M., Tongiorgi E. (2014). Developmental and maintenance defects in Rett syndrome neurons identified by a new mouse staging system in vitro. Front. Cell. Neurosci..

[44] Wahlstedt H., Daniel C., Enstero M., Ohman M. (2009). Large-scale mRNA sequencing determines global regulation of RNA editing during brain development. Genome Res..

[45] Desterro J.M., Keegan L.P., Jaffray E., Hay R.T., O’Connell M.A., Carmo-Fonseca M. (2005). SUMO-1 Modification Alters ADAR1 Editing Activity. Mol. Biol. Cell.

[46] Macbeth M.R., Schubert H.L., Vandemark A.P., Lingam A.T., Hill C.P., Bass B.L. (2005). Inositol hexakisphosphate is bound in the ADAR2 core and required for RNA editing. Science.

[47] Marcucci R., Brindle J., Paro S., Casadio A., Hempel S., Morrice N., Bisso A., Keegan L.P., Del Sal G., O’Connell M.A. (2011). Pin1 and WWP2 regulate *GluR2* Q/R site RNA editing by ADAR2 with opposing effects. EMBO J..

[48] Tariq A., Garncarz W., Handl C., Balik A., Pusch O., Jantsch M.F. (2013). RNA-interacting proteins act as site-specific repressors of ADAR2-mediated RNA editing and fluctuate upon neuronal stimulation. Nucleic Acids Res..

[49] Tan M.H., Li Q., Shanmugam R., Piskol R., Kohler J., Young A.N., Liu K.I., Zhang R., Ramaswami G., Ariyoshi K. (2017). Dynamic landscape and regulation of RNA editing in mammals. Nature.

[50] Filippini A., Bonini D., Lacoux C., Pacini L., Zingariello M., Sancillo L., Bosisio D., Salvi V., Mingardi J., La Via L. (2017). Absence of the fragile X mental retardation protein results in defects of RNA editing of neuronal mRNAs in mouse. RNA Biol..

[51] Hang P.N., Tohda M., Matsumoto K. (2008). Developmental changes in expression and self-editing of adenosine deaminase type 2 pre-mRNA and mRNA in rat brain and cultured cortical neurons. Neurosci. Res..

[52] Cenci C., Barzotti R., Galeano F., Corbelli S., Rota R., Massimi L., Di Rocco C., O’Connell M.A., Gallo A. (2008). Down-regulation of RNA editing in pediatric astrocytomas: ADAR2 editing activity inhibits cell migration and proliferation. J. Biol. Chem..

