# Supplementary material for: Differential Enzymatic Activity of Rat ADAR2 Splicing Variants Is Due to Altered Capability to Interact with RNA in the Deaminase Domain
Source: Genes (Basel). 2018 Feb 8;9(2):79. doi: 10.3390/genes9020079 (PMC5852575; doi:10.3390/genes9020079)
Supplement: Supplementary file 1 [file genes-09-00079-s001.pdf]

Supplementary results

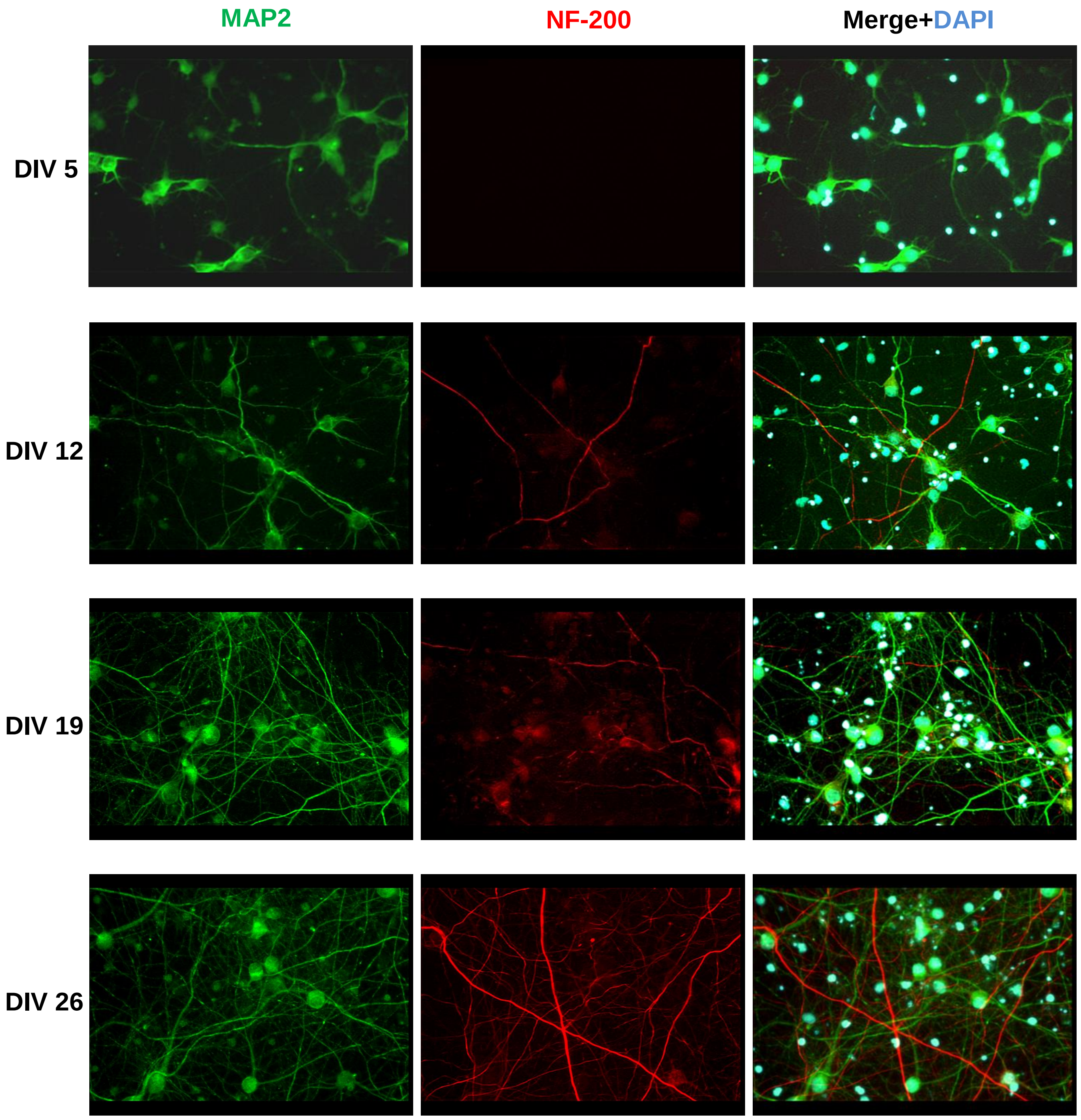

Figure S1: Microtubule Associated Protein (MAP2, green) and Neurofilament 200 (NF-200, red) staining in primary cortical neuron culture at DIV 5-12-19-26. In the merged images the DAPI staining showing nuclei. Magnification 200X. **DIV5 to DIV26 panels show the progressive maturation of neurons expressing an increasing amount of MAP2 and NF-200. The extremely branched dendritic tree with well-defined dendrites and axons**

that exhibited immunoreactivity to NF-200 indicated the generation of healthy mature neurons.

ADAR2 expression during primary cortical neuron cultures maturation

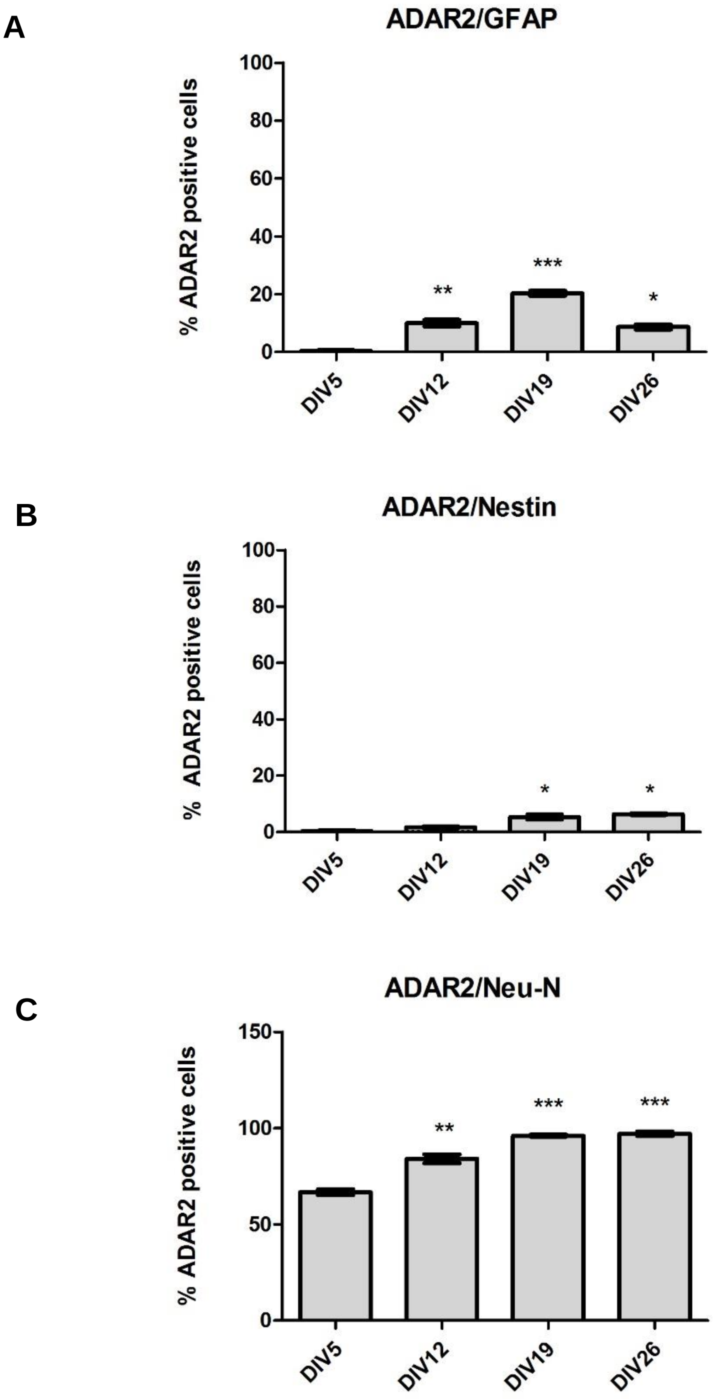

Figure S2: Percentage of GFAP (A), Nestin (B) and Neu-N (C) positive cells expressing ADAR2. Data are presented as means ± SEM of triplicate experiments. Statistical analysis

was performed using one-way ANOVA followed by Bonferroni post hoc test (*p<0.05;**p <0.01; ***p < 0.001). **These results showed that ADAR2 is mainly expressed by cortical neurons.**

Quantification of protein isoforms expression

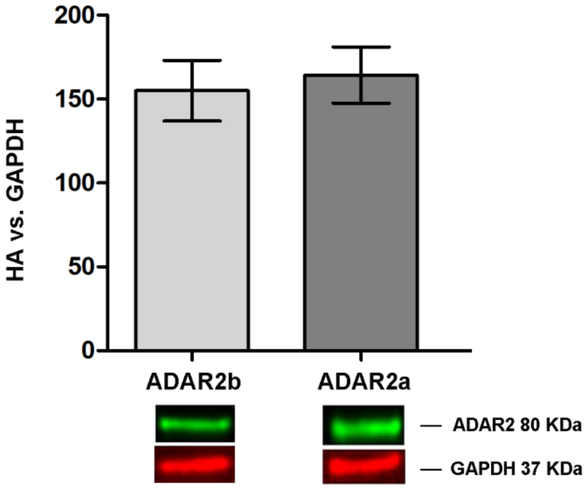

Figure S3: Western blot analysis for rADAR2a and rADAR2b expression. Both proteins are detected through the HA tag. The primary antibody rabbit anti-HA (Sigma-Aldrich, cod: H6908) was used 1:1000 in BSA 5% dissolved in TBST 0.2% 1 h at RT after an overnight incubation with blocking solution BSA 5% in TBST 0.2%. Mouse monoclonal anti-GAPDH (1:10’000, Millipore Billerica, MA 01821; cod: MAB374) was incubated on the membrane overnight at 4° C. IR-Dye® secondary antibodies are both incubated 1 h at RT in TBST 0.2%. Signals were detected using an Odyssey infrared imaging system (LI-COR Biosciences) and quantified using Odyssey version 1.1 (LI-COR Biosciences). Data are presented as means ± SEM of triplicate experiments. Statistical analysis was performed using unpaired T-Test.

No statistically significant difference between ADAR2a and ADAR2b expression following lentivirus infection of primary cortical neurons is detected.

RNA editing levels in PC12 cells overexpressing ADAR2 isoforms

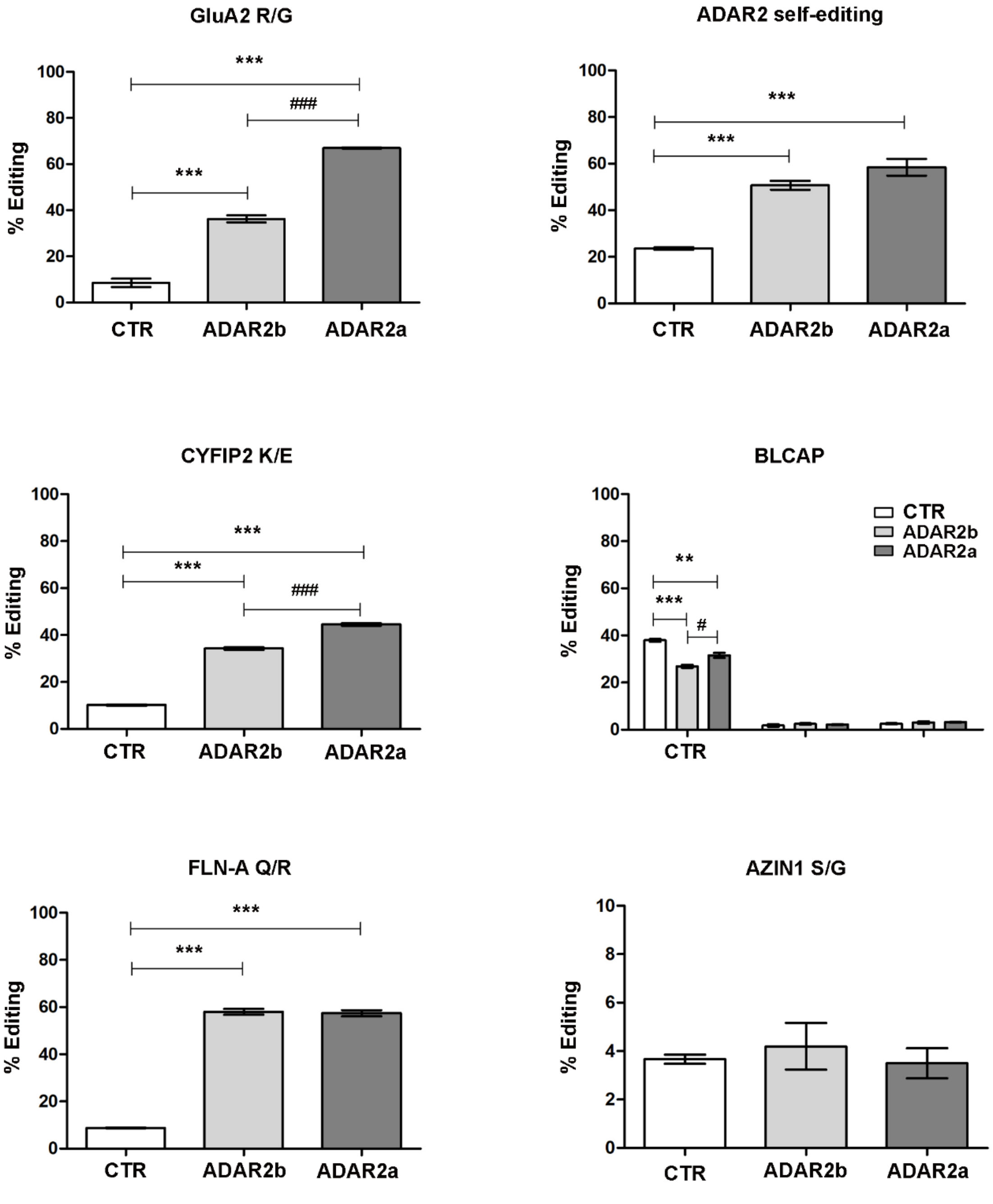

Figure S4: RNA editing levels in PC12 transduced with lentiviral particles carrying ADAR2a or ADAR2b splicing isoforms. Data are presented as means ± SEM of triplicate experiments. White bar: not transduced cells; grey bar: ADAR2b transduced cells; dark grey bar: ADAR2a transduced cells. Statistical analysis was performed using one-way ANOVA followed by

Bonferroni post hoc test (*p<0.05;**p <0.01; ***p < 0.001 vs. CTR; # p<0.05; ## p<0.01; ### p<0.001 vs. ADAR2b).

Increased editing activity was observed for ADAR2a compared to ADAR2b on specific editing sites: GluA2 R/G site (ADAR2b: 36.2±1.53%; ADAR2a: 67±0.2% p<0.001 vs. ADAR2b), CYFIP2 K/E site (ADAR2b: 34.3±0.5%; ADAR2a: 44.5±0.5% p<0.001 vs. ADAR2b).

Regarding BLCAP transcript, overexpression of ADAR2 isoforms affected only Y/C site: both ADAR2 decreased the editing level (CTR: 38±0.6%; ADAR2b: 26.9±0.63% p<0.001, ADAR2a: 31.5±1.12%, p<0.01), but ADAR2a had increased activity if compared to ADAR2b (ADAR2b: 26.9±0.63%, ADAR2a: 31.5±1.12%, p<0.05).

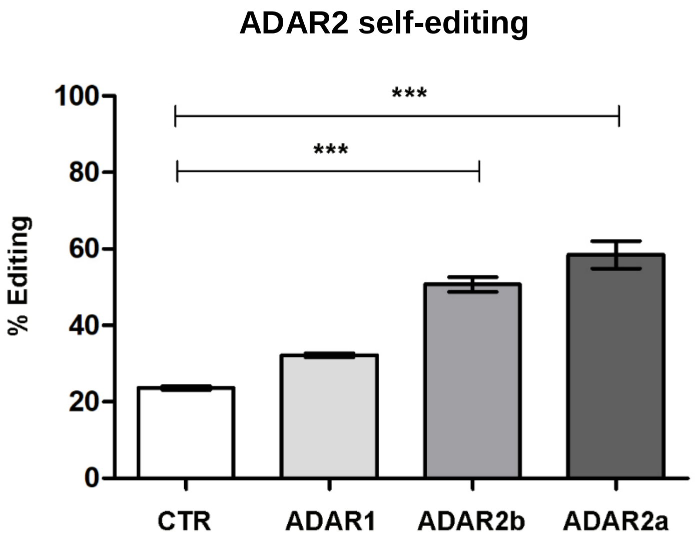

Figure S5: ADAR2 self-editing levels in PC12 transduced with lentiviral particles carrying ADAR1, ADAR2a or ADAR2b splicing isoforms. Data are presented as means ± SEM of triplicate experiment. White bar: not transduced cells. Statistical analysis was performed using one-way ANOVA followed by Bonferroni post hoc test (*p<0.05;**p <0.01; ***p < 0.001 vs. CTR). **No statistically significant increase of ADAR2 self-editing level was observed after ADAR1 lentiviral transduction, meaning that this is an ADAR2 specific editing site.**

A

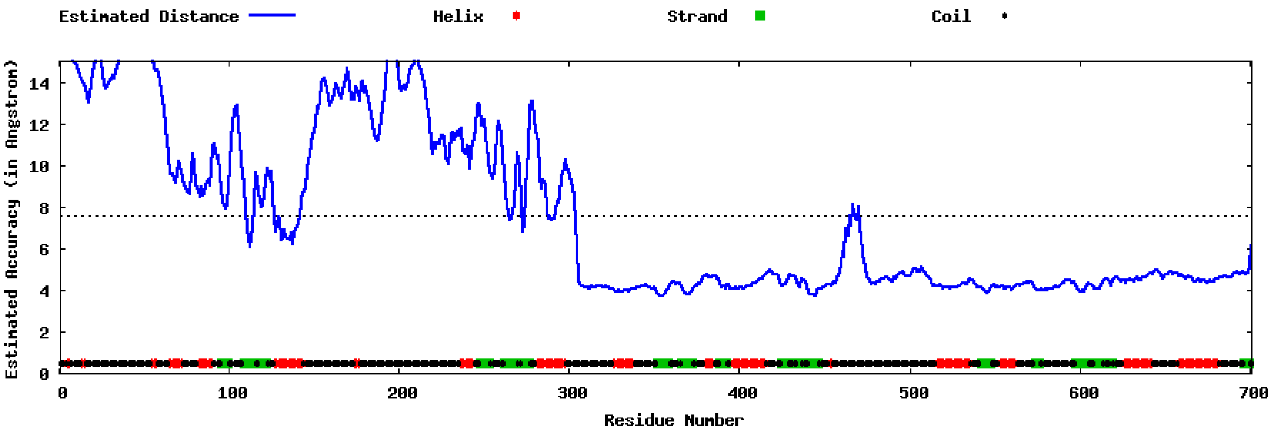

B

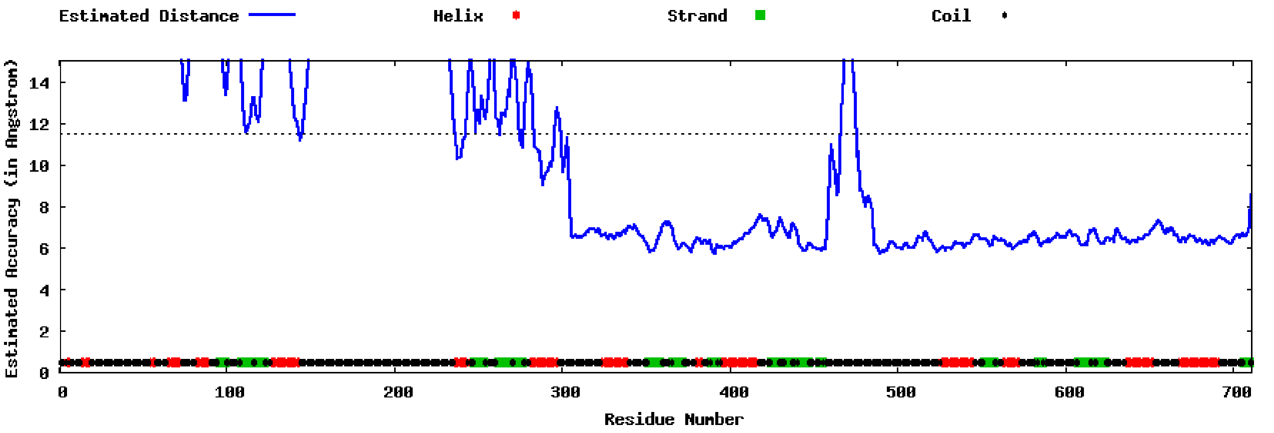

Figure S6: Analysis of local structure quality performed by I-Tasser for the predicted structures of the 2 protein isoforms ADAR2a (A) and ADAR 2b (B). Higher values indicates aminoacid with less accurate positioning in the structure model. **As expected, predictions were more reliable in the C-terminal portions from aa 305 that match the deaminase domain represented in the template structure used for modeling. Only these portions were used for molecular dynamics.**

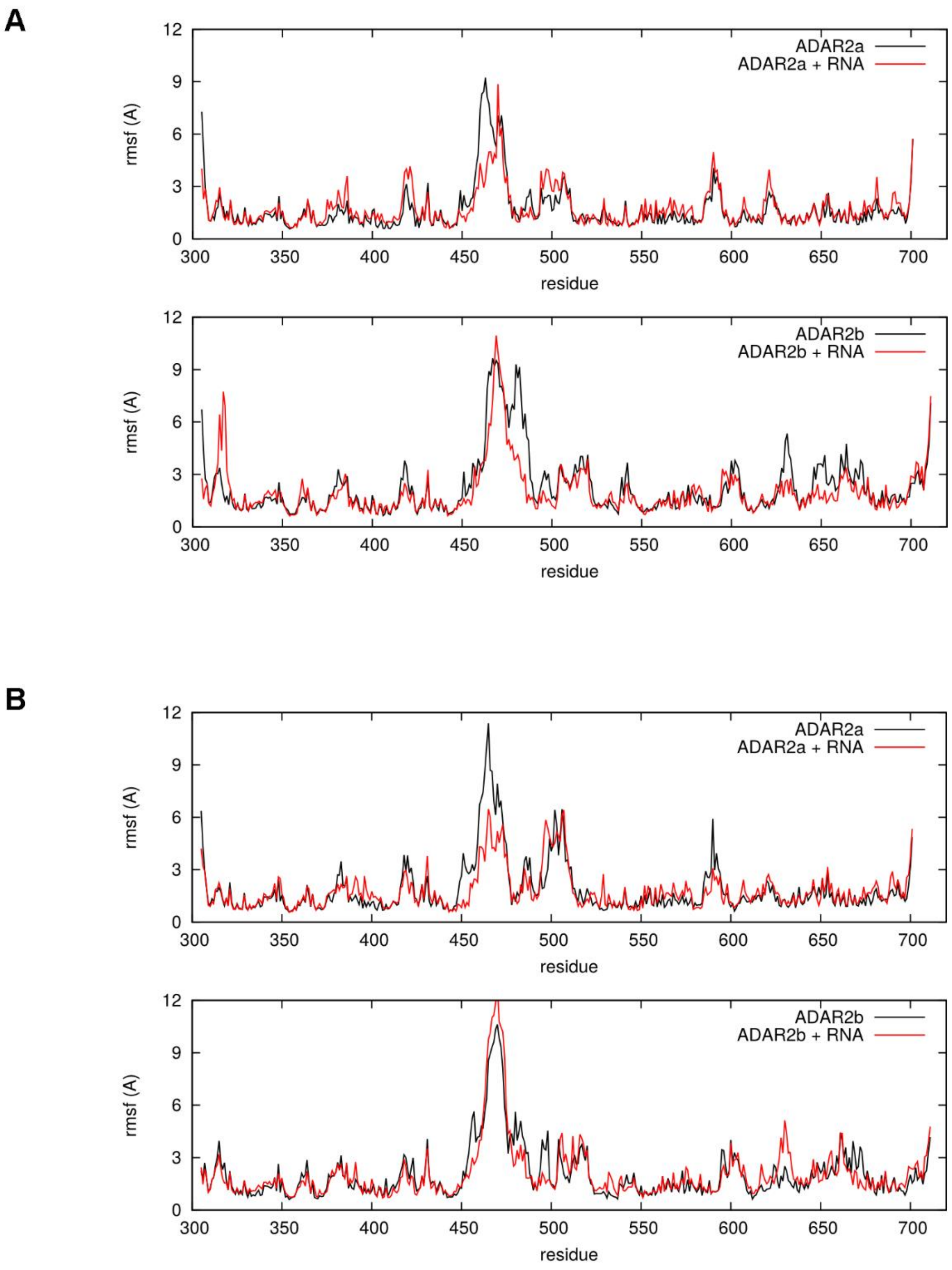

Figure S7: (A) Root mean square fluctuation per residue obtained from the MD simulations of ADAR2a and ADAR2b with and without RNA in the absence of the Zn^{++} ion; (B) root mean square fluctuation per residue obtained from the MD simulations of ADAR2a and ADAR2b with and without RNA in the presence of the Zn^{++} ion.

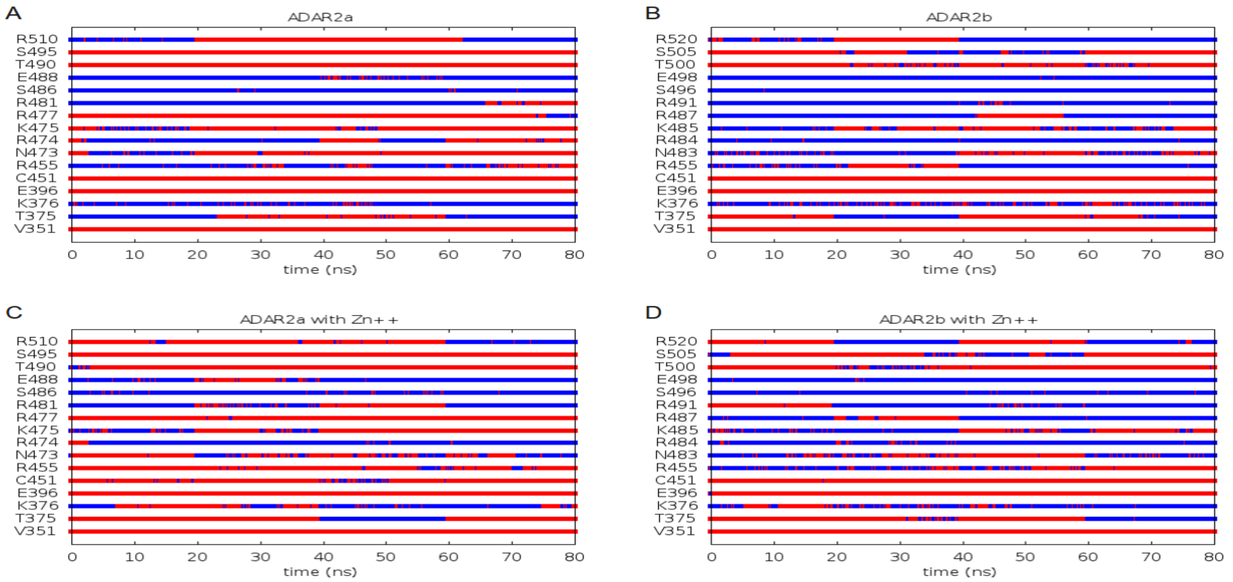

Figure S8: Presence of hydrogen bonds (HB) between selected residues and RNA as a function of time. Red color denotes absence of HB, while blue color denotes presence of at least one HB. Panel A: ADAR2a; panel B: ADAR2b; panel C: ADAR2a with Zn++ ion; panel D: ADAR2b with Zn++ ion.

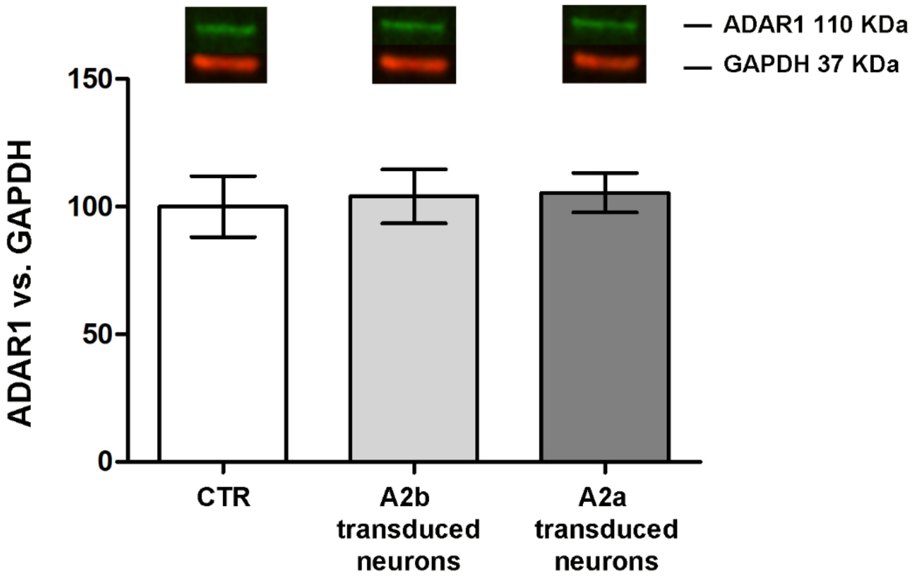

Figure S9: ADAR1 endogenous expression after ADAR2a (A2a) and ADAR2b (A2b) overexpression in neuronal cells. White bar: untransduced neurons; grey bar: ADAR2b transduced neurons; dark grey bar: ADAR2a transduced neurons. **No statistical significant variation was observed.**
